# Extracellular matrix and cellular senescence in venous leg ulcers

**DOI:** 10.1038/s41598-021-99643-9

**Published:** 2021-10-11

**Authors:** Debbie X. E. Lim, Toby Richards, Muholan Kanapathy, Thankiah Sudhaharan, Graham D. Wright, Anthony R. J. Phillips, David L. Becker

**Affiliations:** 1 Lee Kong Chian School of Medicine, Nanyang Technological University Singapore, 11 Mandalay Road, Singapore City, Singapore; 2 Skin Research Institute of Singapore, Agency for Science, Technology and Research, 11 Mandalay Road, Singapore City, Singapore; 3 Faculty of Health and Medical Sciences, University of Western Australia, Perth, Australia; 4 Division of Surgery and Interventional Science, University College London, London, UK; 5 Department of Plastic and Reconstructive Surgery, Royal Free NHS Foundation Trust Hospital, London, UK; 6 Microscopy Platform, Research Support Centre, Agency for Science, Technology and Research, Singapore City, Singapore; 7 School of Biological Sciences, Auckland University, Auckland, New Zealand

**Keywords:** Biomarkers, Diseases, Health care, Medical research, Risk factors, Signs and symptoms

## Abstract

High prevalence of non-healing chronic wounds contributes to a huge healthcare burden across the world. Early treatment interventions for non-healing wounds are vital. It was previously shown that accumulation of 15% or more of senescent cells in a chronic wound edge is an indicator that the wound is unlikely to heal. However, determining the presence of senescent cells would require invasive procedures such as tissue biopsies to be taken. In this study, we found a strong correlation between decreased collagen area and presence of senescent cells in human chronic wounds i.e. venous leg ulcer (VLU), diabetic foot ulcer (DFU) and pressure ulcer (PRU). We also report that the lowest collagen levels were found in VLU patients less than 60 years of age, with a persistent wound of > 24 months. Elevated levels of senescent cells were also found in VLU of males. Second harmonic imaging of collagen at the edge of chronic wounds with a handheld multiphoton device could be used to predict the number of senescent cells, indicating if the wound is on a healing trajectory or not. Our data support the use of collagen imaging in cutaneous wound assessment for a faster and non-invasive method to predict cellular senescence and determining wound trajectory of healing.

## Introduction

Chronic wounds such as venous leg ulcers (VLU), diabetic foot ulcers (DFU), and pressure ulcers (PRU) do not progress through the normal healing process in an orderly and timely manner^1^. Chronic wounds are predominantly seen in the elderly, but can occur in younger patients with diabetes, vascular problems and other underlying medical conditions and are complicated to treat^1^. This creates a major burden in healthcare services costs, which in the United States is estimated to be between $28 to $96 Billion per year, with close to 4.5 million people suffering from chronic wounds^2,3^. With the increasing aging population and growing incidence of diabetes, these ulcers lead to decreased quality of life and loss of function, often ending in lower limb amputation^4^. On average, these ulcers last for 12–13 months, with some persisting for years. In addition, these wounds reoccur in 60–70% of patients with a higher mortality rate of 5 years after amputation surgeries^5^. Successful treatment of chronic wounds remains a challenge, with no reliable indicators of possible non-healing ulcer progression. The duration of this process varies between individuals and can last from 21 days to several years. However, in the diabetic elderly populations the wound healing process can stall at the pro-inflammatory phase resulting in the development of chronic wounds^6,7^. The lack of healing can also be due to underlying peripheral vascular disease or prolonged pressure caused from immobility or wheelchair use^8^.

Chronic wounds are characterized by persistent inflammation, large numbers of senescent cells and excessive proteolytic activity from matrix metalloproteinases (MMPs)^9–14^. The prolonged exposure of chronic wounds to pro-inflammatory cytokines, such as IL-1 and TNF-α, act to stimulate the production of MMPs while inhibiting the synthesis of their regulators, tissue inhibitors of matrix metalloproteinases (TIMPs)^15–17^. When the balance between extracellular matrix (ECM) loss and deposition is disrupted, elevated levels of MMPs and decreased TIMPs causes excessive breakdown of the ECM^18^. The overall inflammatory microenvironment in chronic wounds lead to high oxidative stress, resulting in DNA damage related cell cycle arrest, driving cells into senescence^19^. These cells have decreased proliferation and stress-induced premature senescence^14,15,20–23^. The presence of senescent fibroblasts has been suggested to have a link with compromised healing, with more than 15% senescent fibroblasts indicating poor healing progression^1,2^.

Second harmonic generation imaging (SHG) of ECM collagen has been used to show the loss of ECM in wound edge biopsies of chronic wounds (24). The second harmonic generation of collagen originates from the nonlinear polarization of non-centrosymmetric peptide bonds along the collagen triple helix. Collagen SHG is label-free, remarkably precise and delicate, making it a desired candidate for systematic studies of fibrosis with better reliability than conventional histology.

To date, the relationship between collagen distribution and cellular senescence remains unclear. Here, we document the correlation between ECM composition and the number and location of senescent cells in biopsies of chronic wound edges. This information may serve as a guideline for the use of SHG microscopy to predict the healing trajectory of chronic wounds.

## Materials and methods

### Ethics approval

All wounds with paired arm tissues were ethically approved and obtained from the Western Institutional Review Board (Olympia, WA, USA). Informed consent was obtained from study participants and all methods were performed following the relevant guidelines and regulations. Details of the patients were discussed previously^24^. Ethnic approval for a separate cohort of 26 patients with venous conditions was obtained at University College London and Royal Free Hospital (London, UK)^25^. These tissues were obtained from the National Research Ethics Service Committee London—South East (11/LO/1483) and Nanyang Technological University Institutional Review Board (IRB-2015-05-003). Detailed patient information was included earlier in^25^.

### Masson trichrome histological stain

Frozen sections were defrosted and immersed in phosphate buffered saline (PBS) to dissolve excess optimal cutting temperature (OCT) compound. They were then transferred to Bouin’s solution (Sigma-Aldrich, UK—HT10132) for incubation overnight. Sections were washed under running tap water for 10 min prior to two changes of deionized (DI) water before staining with freshly made Weigert’s Iron Haematoxylin (Merck, UK—1.15973) for 5 min, followed by 3 min of running tap water and transferred to Biebrich Scarlet working solution [1% Ponceau BS (Merck, UK—B6008) in DI water; 1% Fuchsin acid (Sigma-Aldrich, UK—F8129) in DI water and 1% glacial acetic acid (Merck, UK—A6283)] for 2.5 min. Sections were briefly rinsed in 1% acetic acid until the solution ran clear. They were then differentiated in 5% phosphomolybdic-tungstic acid working solution [10% phosphomolybdic acid solution (Sigma, Aldrich, UK—79560) in DI water; 10% phosphotungstic acid (Sigma-Aldrich, UK—P4006) solution in DI water] for 30 min. Collagen was stained with 2% light green (Sigma-Aldrich, UK—L5382) for 20 min and rinsed with DI water. The tissues were left to dry at room temperature (R.T) for at least 45 min before mounting with limonene. Staining: Collagen—green; nuclei—black; muscle fibres and cytoplasm—red.

### Senescence histological stain

The slides were air-dried at R.T for at least 1 h and washed 2 times in 1 × PBS for 5 min prior to starting the staining. 200 µl of senescent cell staining kit [1 ml of 10 × staining solution; 125 µl of Reagent B; 125 µl of Reagent C; 250 µl of X-gal solution in 8.5 ml of Millipore water, filter-sterilized (Sigma-Aldrich, UK—CS0030) was added to each tissue section and incubated for 24 h at 37 °C. The slides were washed in PBS once for 5 min and counterstained for nuclei with nuclear fast red solution (Abcam, Cambridge, UK—ab246831) for 10 s. The tissues were then placed into quick washes, 2 times in DI water prior to dehydration through alcohol baths and clearing with clearene. Tissues were mounted with limonene. Staining: Senescent cells—green/blue; Nuclei—pink.

### Haematoxylin and eosin (H&E) stain

All tissue sections included in this study were concurrently stained with hematoxylin and eosin. Excess OCT was removed by washing slides 3 times in 1 × PBS for 5 min each. The slides were put through a Leica autostainer XL (Leica, Germany) set to the staining protocol: Hematoxylin—1 min, wash—3 min, acid alcohol—3 s, wash—2 min, Scott’s tap water—5 s, wash—2 min, 70% ethanol—2 min, eosin—30 s followed by a series of alcohol baths and clearing with clearene for dehydration. Slides were mounted with limonene.

### Image acquisition

All slides stained via Masson Trichrome and X-gal senescence kit were imaged using the 20 × objective in the brightfield mode on an AXIO Scan.Z1 slide scanner (Zeiss, Germany). Four specific regions (two from the high senescence and two from the low senescence regions; 1200 × 1000 μm) for each tissue were selected and cropped using ZEN lite 2.3 software (Zeiss, Germany). The SHG imaging was performed using LaVision TriM Scope II (Miltenyi Biotech, Germany) inverted (Nikon Ti) microscope equipped with Two-photon Chameleon (Coherent) Ti:Sapphire tunable laser (680–1080 nm), Zeiss W Plan- Apochromat 20 ×/1.0 water dipping objective and GaAsp detectors. The SHG collagen signal was excited at 880 nm (~ 3100 mW laser power) and detected with 420–460 nm band pass emission filter by placing 595 dichroic mirror in the emission light path. Mosaic SHG image data acquisition was performed using LaVision software, Inspector Pro 5.1.371 version with a tile overlap of 5%. Images having 6 × 6 grid size of 463 × 463 μm (Arm) and 11 × 8 grid size of 477 × 477 μm (VLU) were generated. 2D images were acquired by having a scanning frequency of 400 Hz with a dwell time of 4.0 and 2.0 respectively. The mosaic images generated were stitched using Image J version Java 1.6.0_65 (32-bit) (National Institutes of Health, Bethesda, Maryland, USA).

### Quantification of collagen fibres by Image J

All images were quantified using Image J. The four steps to collagen quantification described in^26^ were adopted in our analysis with defined changes. Colour deconvolution with vectors for Masson Trichrome was selected, and Colour 3 (green) was selected for collagen measurement in each image. Gaussian Blur with sigma = 4.00 was performed, and threshold “Li” was utilized at a value of 0, 217 (min, max). The images were set to true black background. Analyse particles displaying summarized measurements were selected and results were extrapolated.

### Quantification of senescent cells by Image J

All images were quantified by performing ‘Split Channels’ function. The red channel derived was utilized to quantify cell senescence while the green channel was utilized for overall nuclear counting. The auto threshold “Shanbhag” was selected at a value of 0, 225 (min, max) with background set to false. The images were converted to mask and watershed once. Analyse particles displaying summarized measurements of cell size from 100 to infinity was selected. For nuclear counting, the auto threshold “Li” was applied at a value of 0, 220 (min, max) with background set to false on the green channel. The images were also converted to mask and watershed once. Analyse particles displaying summarized measurements of cell size from 200 to infinity was selected.

### Statistics

All statistical analysis was carried out on GraphPad Prism version 7.00 for MAC, GraphPad Prism software (La Jolla, California, USA). The data were first tested for normality using the Kolmogorov-Smirnoff test, permitting parametric analysis. Data was then analysed using Two-Way ANOVA to determine interaction between independent variables and factors compared, followed by Tukey’s post ad-hoc multiple comparison test. Significance was considered at values of *p* ≤ 0.05. Regression analysis to determine correlation was performed using Microsoft Excel version 14.1.0 for MAC and the “Add Trendline” function to display R-squared value and equation without setting an intercept.

## Ethics approval

All chronic leg and foot wound and normal arm tissue was sourced in the USA (Western Institutional Review Board, 3535 Seventh Avenue, Olympia, WA 98502-5010). Ethic approval for tissues with venous conditions (CEAP 2–6) were obtained from the National Research Ethnics Service Committee London—South East (11/LO/1483) and Nanyang Technological University Institutional Review Board (IRB-2019-01-041).

## Results

Tissues from 15 VLU patients were evaluated, with an average age of 59 (31–79 years). The cohort was 63% male, with average wound age of 6.75 months (1–108 months) and 56% were diabetic. Information on wound age for 2 samples were unavailable and were excluded from wound age analysis. Tissues from 5 DFU male patients were examined, with an average age of 59 (48–82 years) and average wound age of 4 months (1.5–26). PRU cohort contained tissue from 5 patients, with an average age of 60 (34–82). 80% of the cohort were males with average wound age of 8.4 months (4–48) and 40% of them were diabetic. C2–C6 tissues were added to the study to evaluate collagen area across different venous stages. All samples were taken from wound edge/below knee (WE/BK) with paired above knee (AK) tissues. Tissues in the C0 stage (no visible sign of disease) was not included. Patients under this group were 53.9% female and 46.1% male, with average age of 58.2 and 62.9 respectively.

### Examination of extracellular matrix and senescent cells in human chronic wounds

**Figure 1:**
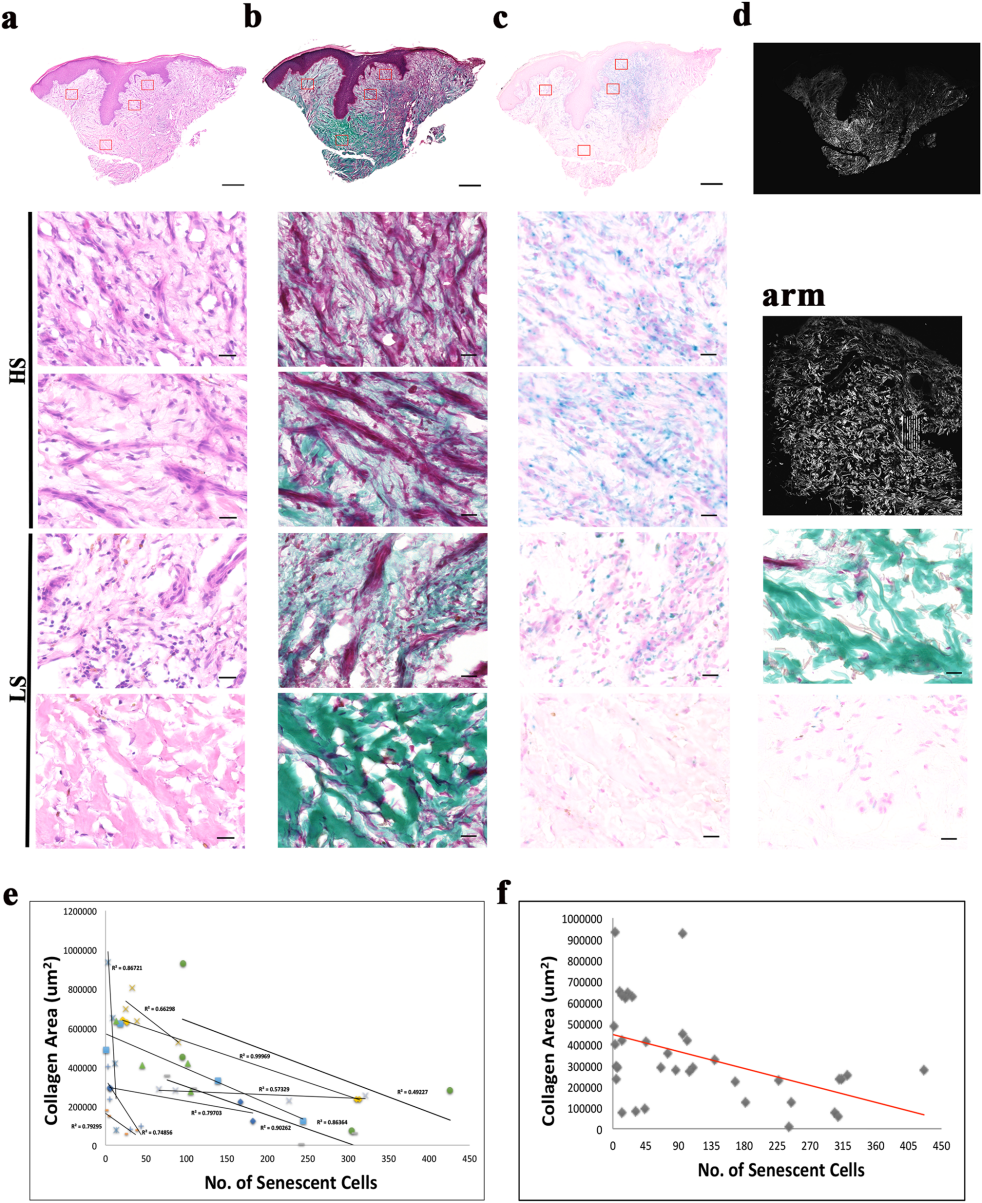
Staining of nuclei, collagen and senescent cells of venous leg ulcer biopsies revealed a strong link between presence of senescent cells and collagen area. (**a**) Haematoxylin and eosin staining of nuclei (purple) and extracellular proteins (pink) in a venous leg ulcer. Montage of high-power images [1200 (W) × 1000 (H) μm] of an 8 μM cryosection thick—two from high senescence (HS) regions and two from low senescence (LS) regions of a venous leg ulcer taken using × 20 objective lens. (**b**) MT staining collagen (green), muscle and keratin (red) and cytoplasm (pink/red) in a sister section. Montage followed by (**c**) X-gal staining as a marker for senescent cells (senescent cells (blue), nuclei and cytoplasm (pink). Scale bar: 500 μm (montage) and 20 μm (high power). (**d**) SHG imaging of collagen (white) in × 20 for VLU and arm tissue. Scale bar: 200 μm. MT and X-gal images of the arm tissue are also shown. (**e**) Regression plots of collagen area (x-axis) against senescent cell number (y-axis) of individual patients of the VLU cohort. (**f**) Single regression plot of the VLU cohort.

**Figure 2:**
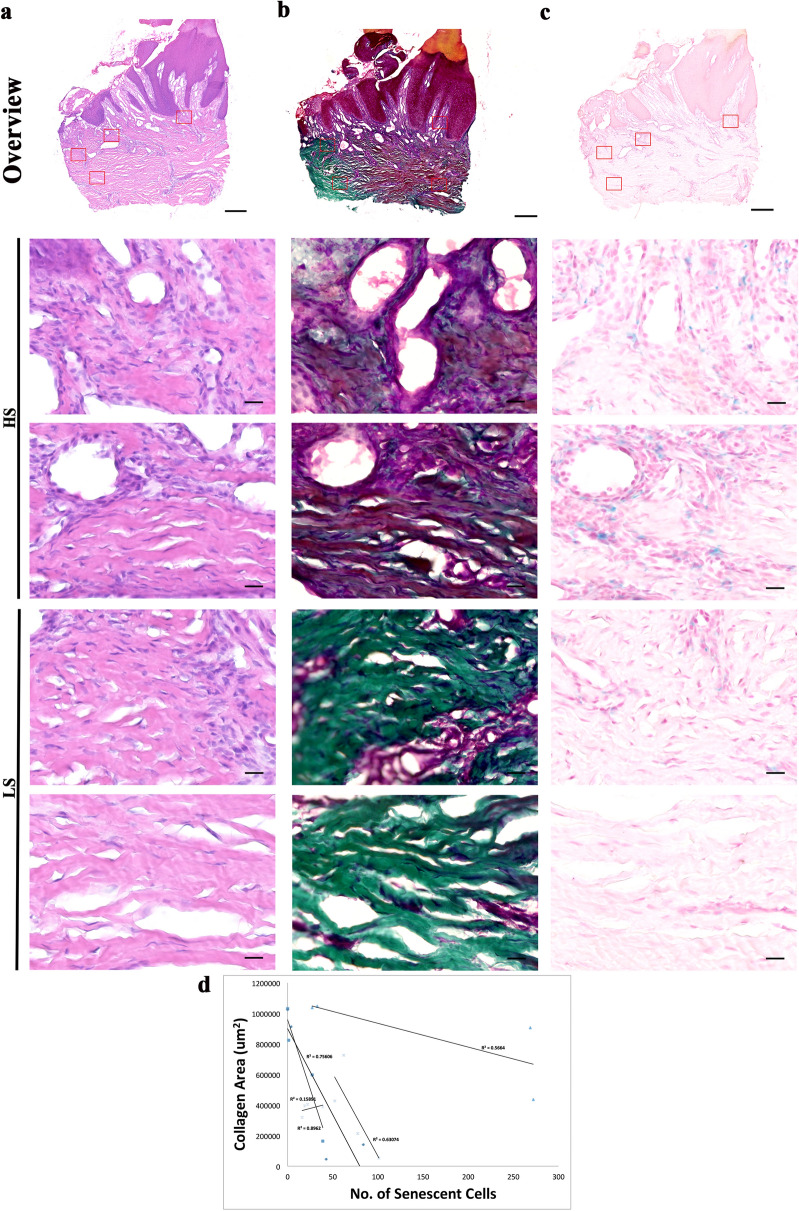
Staining of nuclei, collagen and senescent cells of diabetic foot ulcer biopsies revealed a strong link between presence of senescent cells and collagen area. (**a**) Haematoxylin and eosin staining of nuclei (purple) and extracellular matrix proteins (pink) in a diabetic foot ulcer. Montage of high-power images [1200 (W) × 1000 (H) μm] of an 8 μM thick cryosections—two from high senescence (HS) regions and two from low senescence (LS) regions of a diabetic foot ulcer taken using × 20 objective lens. (**b**) MT staining collagen (green), muscle and keratin (red) and cytoplasm (pink/red) in a sister section. Montage followed by (**c**) X-gal staining as a marker for senescent cells (senescent cells (blue), nuclei and cytoplasm (pink). Scale bars, montage 500 μm and high power 20 μm. (**d**) Regression plots of collagen area (x-axis) against senescent cell number (y-axis) of individual patient of the DFU cohort.

**Figure 3:**
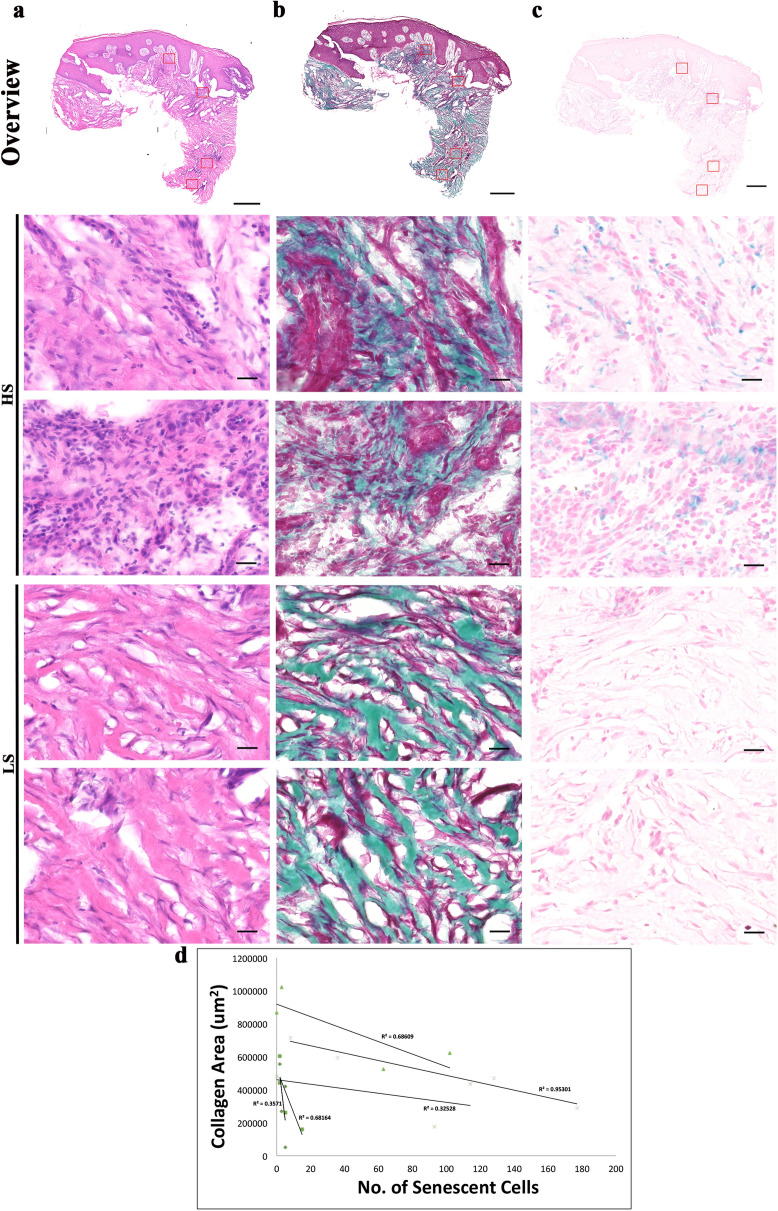
Staining of nuclei, collagen and senescent cells of pressure ulcer biopsies revealed a strong link between presence of senescent cells and collagen area. (**a**) Haematoxylin and eosin staining of nuclei (purple) and extracellular matrix proteins (pink) in a pressure ulcer. Montage of high-power images [1200 (W) × 1000 (H) μm] of an 8 μM thick cryosections—two from high senescence (HS) regions and two from low senescence (LS) regions of a pressure ulcer taken using × 20 objective lens. (**b**) MT staining collagen (green), muscle and keratin (red) and cytoplasm (pink/red) in a sister section. Montage followed by (**c**) X-gal staining as a marker for senescent cells (senescent cells (blue), nuclei and cytoplasm (pink). Scale bars, montage 500 μm and high power 20 μm. (**d**) Regression plots of collagen area (x-axis) against senescent cell number (y-axis) of individual patients of the PRU cohort.

To study the correlation between extracellular matrix composition and the presence of senescent cells, serial sections of the three types of chronic wound edge tissues and their respective intact arm tissues were stained with MT or X-gal (Figs. 1, 2, 3). To examine links between collagen levels and senescence, two regions of interests (ROIs) from areas with high senescence (HS) and two ROIs from regions with low senescence (LS) were selected from each chronic wound tissue. ROIs on the arm tissues were randomly selected. Selected ROIs were kept consistent as far as possible to permit quantification and comparison within each sample. Regions where thick bundles of extracellular matrix fibrils remained in the arm tissue, senescent cells were not present. H&E staining of the respective wounds are shown in Figs. 1a, 2a and 3a. In contrast, in chronic wounds where loss of thick collagen fibrils was seen (Figs. 1b, 2b and 3b), high levels of senescent cells were observed (Figs. 1c, 2c and 3c). Collagen SHG images of VLU and paired arm were shown in Fig. 1d. All control arm skin tissues stained minimally with X-gal, indicative that senescent cells were absent in these healthy skin tissues. Regression plots showing the relationship between collagen area and senescent cell numbers reported strong to very strong negative correlation (R values = 0.31–0.98) in all chronic wound types (Fig. 1e, 2d, 3d), except for one DFU patient, where the R value was 0.005 (Fig. 2d). Single regression plot was shown for VLU to show the heterogeneity of the cohort (Fig. 1f).

### Effects of wound age on collagen decrease in the VLU cohort

**Figure 4:**
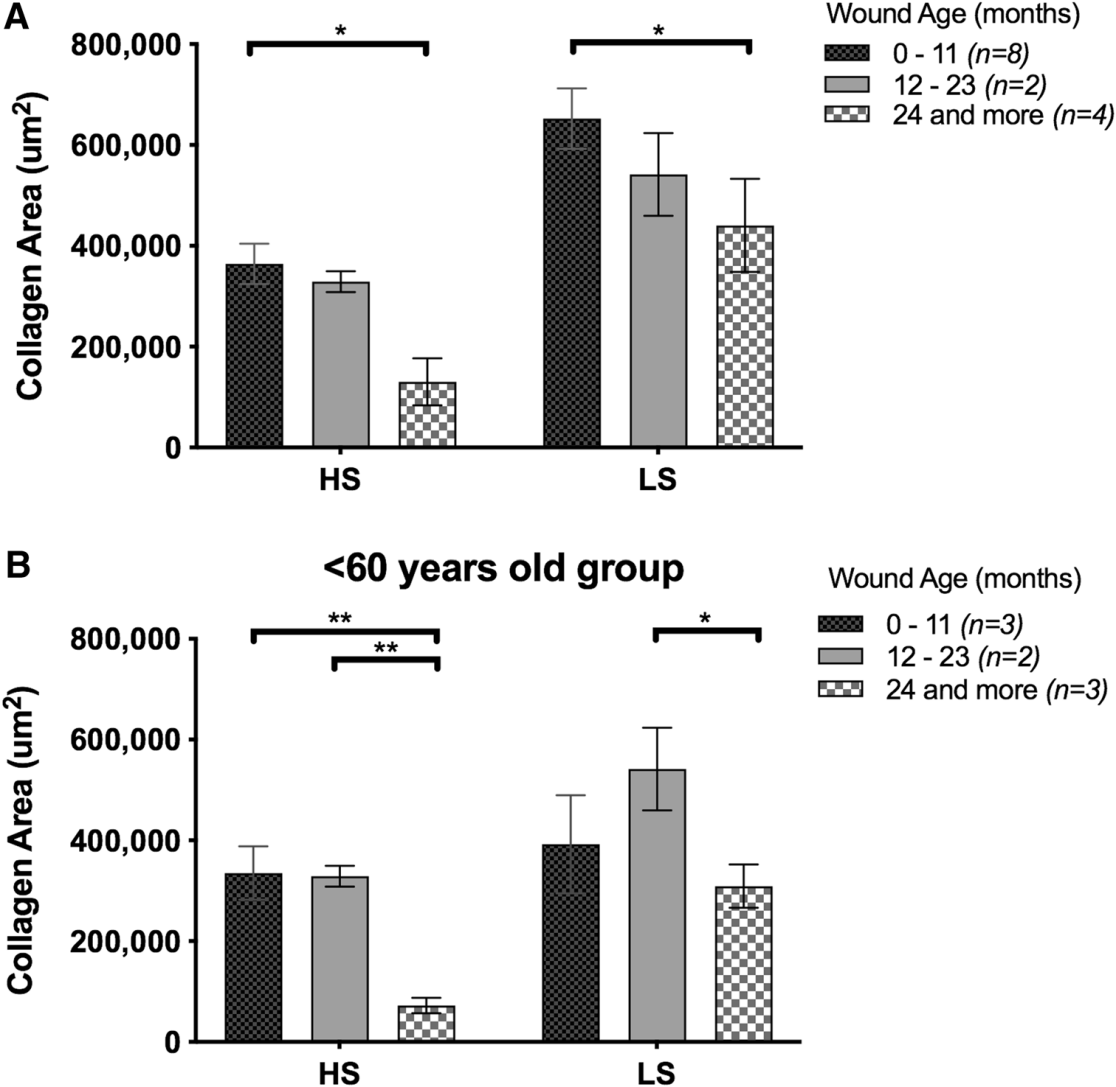
Collagen area is wound-age dependent in the VLU cohort. (**a**) Collagen area of VLU patients were categorized based on the duration of each wound: 0–11 (*n* = 8), 12–23 (*n* = 2) and more than 24 months (*n* = 4). (**b**) Collagen area of less than 60 years old VLU patients: 0–11 (*n* = 3), 12–23 (*n* = 2) and more than 24 months (*n* = 3) at point of biopsy and grouped based on wound duration. Data shown as mean ± SEM. *p < 0.05, **p < 0.001.

To examine if prolonged wound age corresponded to decreased collagen area, the VLU patients were categorized based on their respective wound durations—0–11, 12–23 and more than 24 months. It was apparent that the longer a wound persists, the greater the extent of collagen loss. Patients with wounds of 24 months or longer showed statistically lower collagen area compared to those with wounds of 11 months or less (Fig. 4). To investigate if reduced collagen levels is also dependent on chronological age, we split the cohort into 2 groups—under 60 (Fig. 4a), and over 60 (early old age) (Fig. 4b). We found that patients under 60 showed statistically lower collagen area with longer wound age (more than 24 months) as compared to 23 months or less. This suggests that wound duration is a better indication of collagen area (Fig. 4b).

### Effects of gender on increased population of senescent cells in the VLU cohort

**Figure 5:**
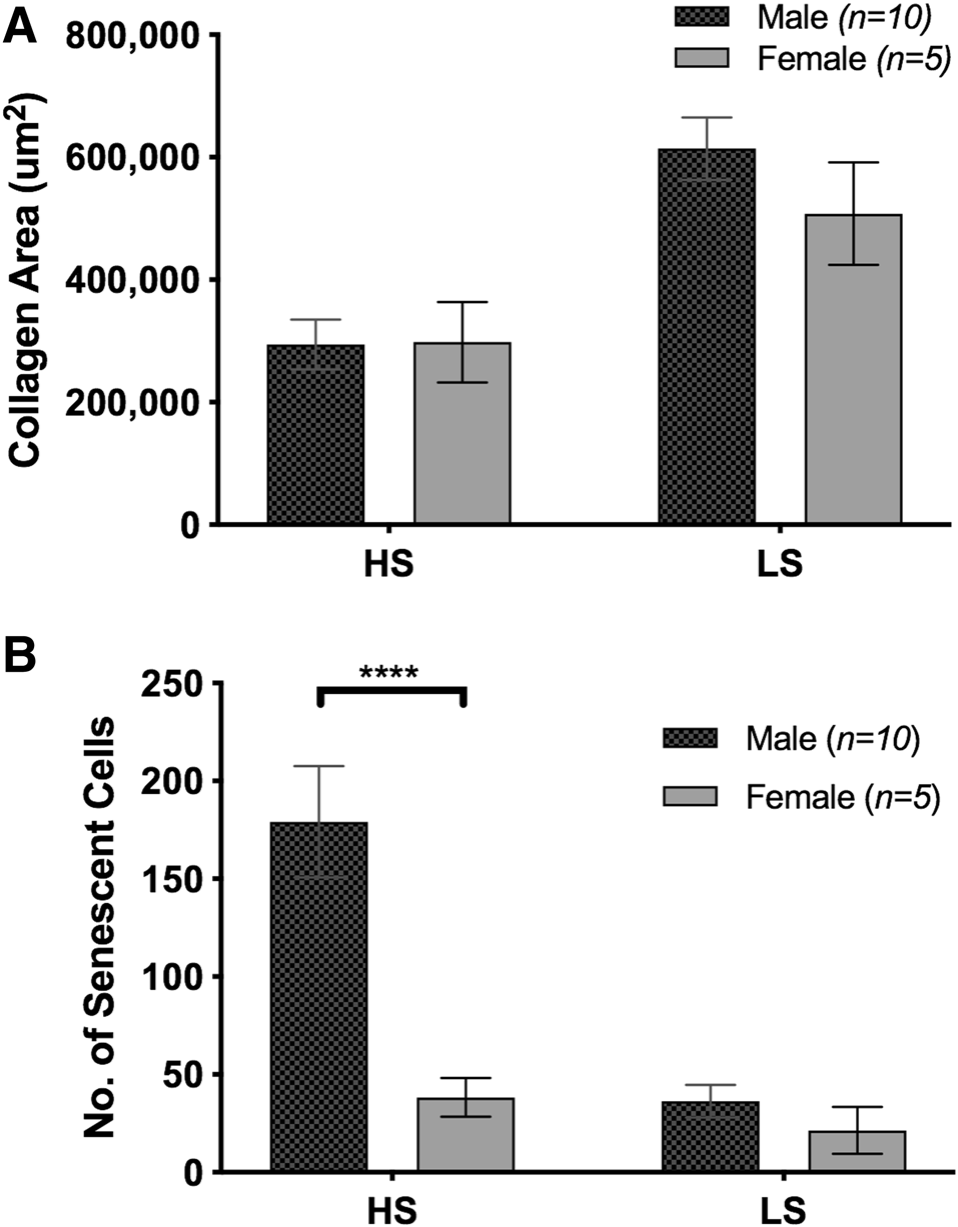
Higher number of senescent cells present in males, with no statistical difference in collagen area across genders in the VLU cohort. Graphs showing (**a**) collagen area of both males and females quantified in regions of high and low senescence [Male (*n* = 10); Female (*n* = 6)] (**b**) senescent cells quantification of males and females in the VLU cohort. Data shown as mean ± SEM. ****p < 0.0001.

We investigated the effects of gender on collagen area but found no statistical difference between genders (Fig. 5a). Interestingly, we found that there was a significant increase of senescence in males compared to females in the VLU cohort (Fig. 5b), in both HS and LS regions.

### Projection of senescent cell numbers and collagen area for early diagnosis of non-healing wounds

**Figure 6:**
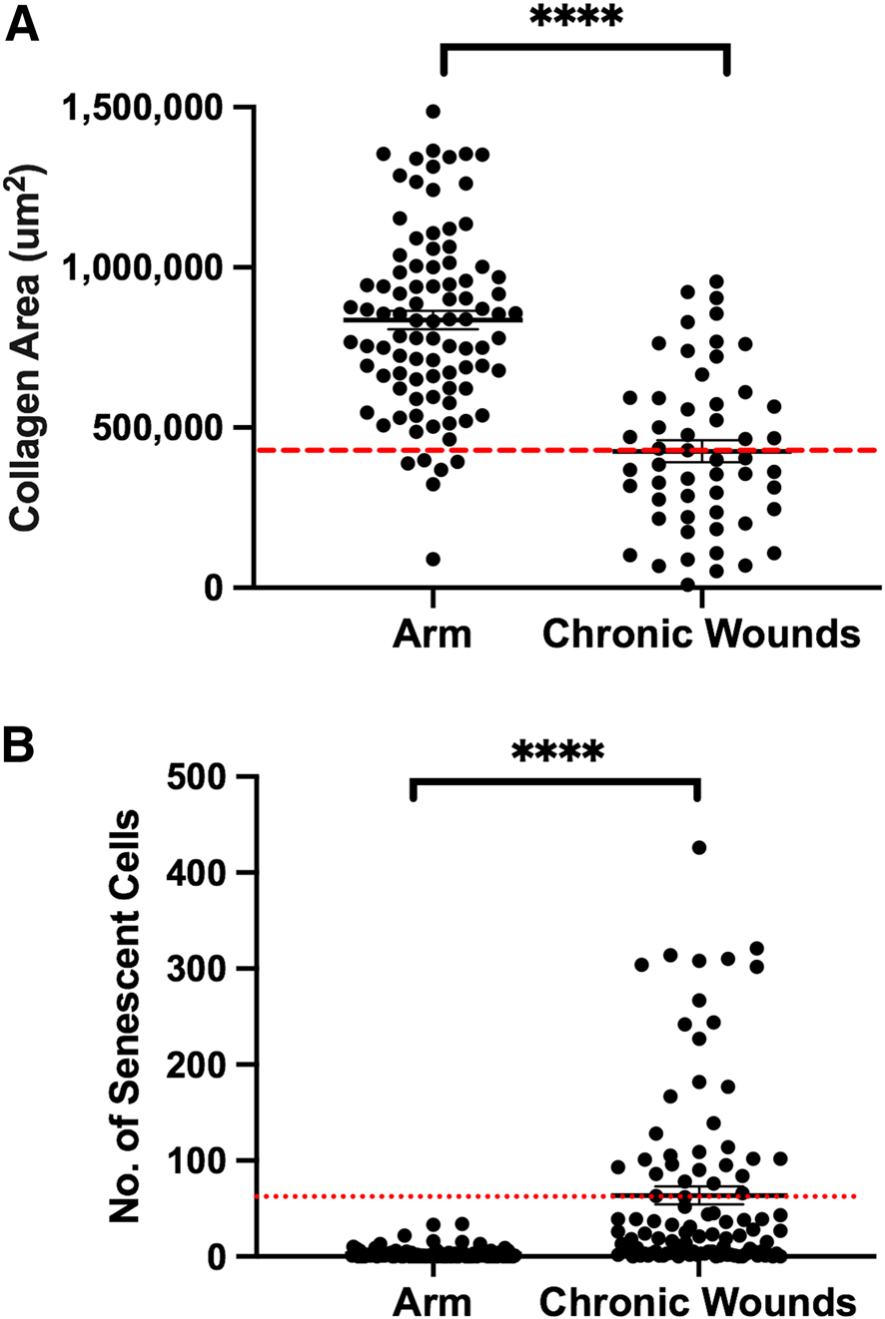
Collagen area and senescent cell numbers of all intact skins and chronic wounds in this study. Graphs showing (**a**) collagen area of arm and chronic wound tissues (**b**) senescent cell numbers of arm and chronic wounds respectively (*n* = 26). Threshold of collagen and senescent cells denoted by red dashed lines across the graphs. Data shown as mean ± SEM. ****p < 0.0001.

Collagen levels in all chronic wound edges were plotted alongside that of the paired control tissues. All control tissues fall between 500,000–1,200,000 µm^2^, with an average of 830,000 µm^2^. However, collagen area measured in the chronic wounds averaged of 420,000 µm^2^, approximately 50% less than that of the arm tissues (*p* < 0.0001) (Fig. 6a). The average number of senescent cells across control tissues was 3 per field of view, whilst chronic wound tissues averaged 64 per field of view (Fig. 6b). 15% of total nuclei count (approximately 300 cells per field of view) in most wounds is approximately 45 cells (Supp. S1), extrapolating to a collagen area of 400,000 µm^2^ based on the regression plot in Fig. 1f. This information could serve as a benchmark for clinicians to determine if a wound is on a healing trajectory or not.

### Collagen levels across clinically classified venous diseases

**Figure 7:**
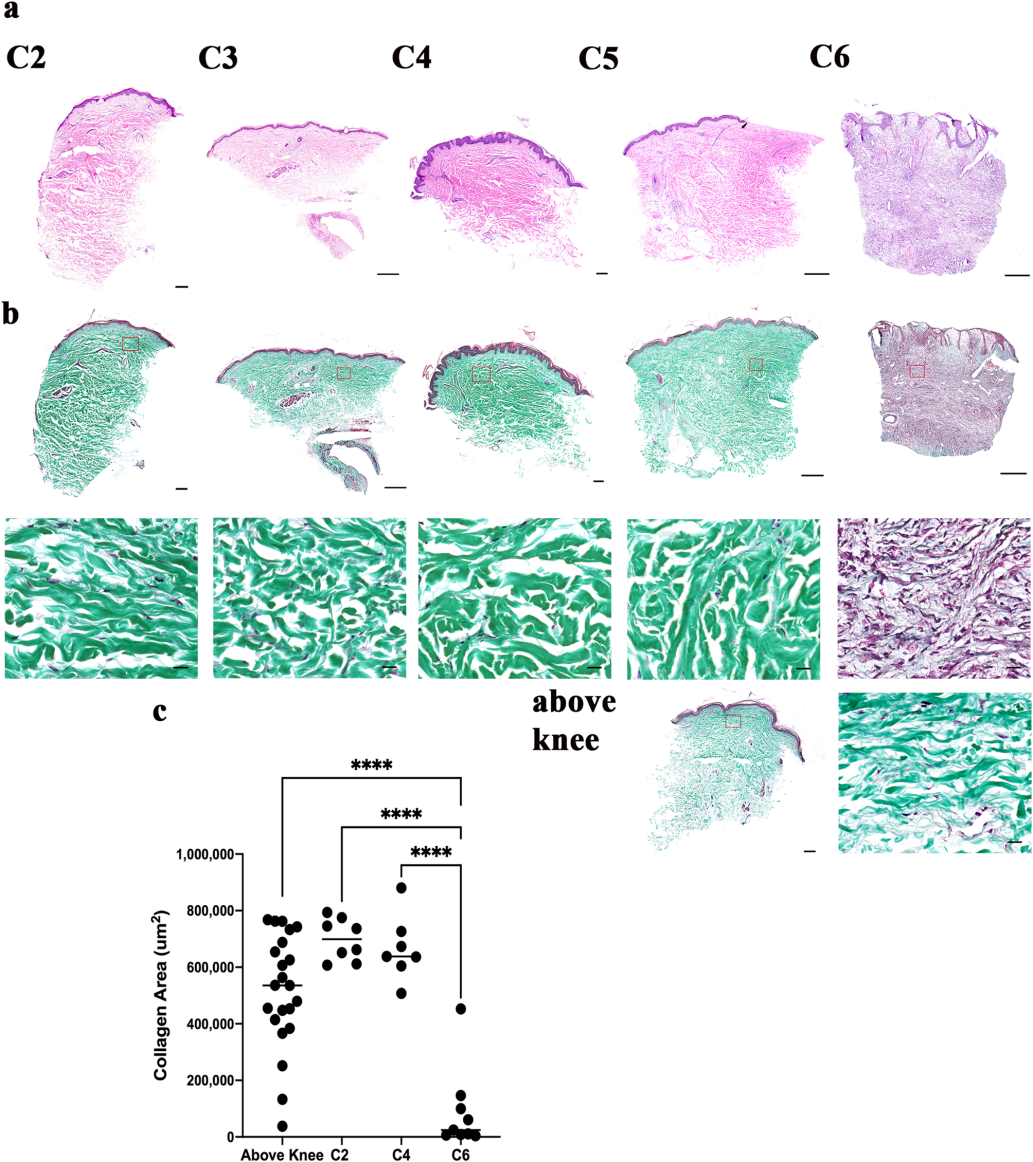
Collagen distribution across venous disease stages. (**a**) H&E staining of C2 to C6 venous disease tissues classified under CEAP. (**b**) MT staining of C2–C6 tissues showing no significant difference in collagen distribution from stage C2–C5, but extended collagen loss at C6. An example of the above knee tissue is also shown. (**c**) Quantification of absolute collagen area across the different venous stages.

To better understand at which venous disease stage collagen starts to degrade, a separate set of tissues classified based on CEAP was examined. Patients were classified into one of these stages—C2 (varicose veins), C3 (edema), C4 (pigmentation, lipodermatosclerosis), C5 (healed venous ulcer) or C6 (venous leg ulcer) (Fig. 7a). No significant change in collagen levels in WE/BK samples were detected across C2–C5 but a drastic drop in collagen levels was seen when wounds progress to C6 (*p* < 0.0001) (Fig. 7b,c).

## Discussion

The complexity of skin wounds encountered in the clinic is one of the main causes for poor diagnosis, resulting in non-healing wounds over time. Factors such as patient age, health status, wound duration, and co-morbidities contribute to wound diversity. This calls for better understanding of patient heterogeneity in wound healing, and to develop new strategies for accurate and timely prognosis.

Here, we investigated the relationship between collagen distribution and accumulation of senescent cells to see if it gives insight to wound progression. Loss of collagen has previously been described in all major chronic wound types^24^. It has also been suggested that accumulation of more than 15% of senescent cells in the wound edge the wound is unlikely to heal^27^. By conducting conventional histological analysis of collagen distribution and cellular senescence, our studies revealed that wounds with extensive collagen loss also contain a large number of senescent cells in the same location.

Exploring relations between patients background such as chronological age, wound period and gender, we found evidence for gender-dependent accumulation of senescent cells and wound-age dependent collagen loss in the VLU cohort. In impaired wound healing, the recruitment of fibroblasts and deposition of collagen are suppressed^28,29^. The elevation of MMPs and suppression of their inhibitors, TIMPs, also decreases the collagen levels in chronic wounds^13,30^. This is evident in our cohort where collagen areas of these tissues were > 50% lower than control skin tissues in all chronic wound types.

Senescent cells are quiescent by nature, but they remain metabolically active^31^. The key feature of senescent fibroblasts is the senescence-associated secretory phenotype (SASP) characterized by upregulated cytokines, ECM-degrading enzymes such as MMPs as well as downregulated expression of ECM components (i.e., collagen)^32,33^. This could explain the negative regression between increased senescent cells and reduced collagen area in chronic wounds observed in our study.

We found reduced collagen area in chronic wounds of more than 24 months. There has been a plethora of reports showing that ulcer duration is negatively related to healing^33–37^. When wounds persist for more than 36 months, they are 17–40% less likely to heal than wounds of less than 1 month^37–42^. Franks et al. reported ulcer duration of more than 6 months having independent risk factors for failure of wound closure^39^. The duration of non-healing wounds was found to be inversely proportional to collagen levels in this study. Thus, collagen levels could serve as an indication of the likely healing trajectory of a wound. Elevated collagen loss was also seen in patients less than 60 years of age, suggesting that collagen loss in the VLU cohort could occur in younger patients with longer wound durations.

Several studies have shown that in instances where elderly men have elevated testosterone levels, they also showed delayed wound healing^43^. Male gender was one of the predisposing factors to poor wound healing, in venous leg ulcers. In our study, male VLU patients had a significantly higher number of senescent cells compared to the females. Although males reported higher mean age (60.8) than the females (43.8), the trend of high numbers of senescent cells was gender-dependent rather than age-dependent, as young males in the cohort also had high numbers of senescent cells. Female estrogen influences the mitochondria in several cell types and is thought to protect the skin from accumulating mitochondrial oxidative damage and mitochondrial-driven senescence^44^. The female mitochondria produce less reactive oxygen species (ROS) through higher expression and activity of antioxidant enzyme systems compared to males^45^. Given that oxidative stress is one of the many causes of cell senescence and mitochondria are the main source of ROS, the production of less ROS in females could reflect fewer senescent cells. In other studies, a high rate of reduction in T cell subpopulations and decline of cytokines IL-6 and IL-10 (immune-inflammatory suppressor) in males^46^ also resulted in a rise in senescence.

It has been reported in our previous work that Connexin 43, a biomarker for poor wound healing, is increasingly expressed in lower limbs from varicose veins through to ulceration^25^. Here, collagen levels in venous leg disease with chronic venous insufficiency CEAP stages from C2 to C6 non-healing ulcer were examined. We found consistent collagen content in varicose veins through venous eczema tissues (C2–C4), but collagen decreased drastically in an open chronic wound, C6. Collagen seems to be deposited once again when the wound healed (C5) but more sample numbers are needed to better support this finding. Histological representations of varicose vein tissues reveal increased collagen Type I, with decreased Type III^47^.

The use of SHG imaging in biomedicine has significantly increased in recent years. SHG imaging of collagen in rat-tendon cryosections was reported in our previous study^48^, discussing the potential use of SHG for wound healing assessment. The ability of SHG to detect collagen Types I and III makes it a powerful diagnostic technique that could be transferred to the clinic to indicate the healing trajectory of chronic wounds. In recent years, handheld multiphoton devices have been developed for clinical imaging of thick and live tissue such as human skin at reasonable speed^49^. SHG imaging of collagen could be the next generation of wound monitoring methods in clinics.

We identified two potential limitations within this study. Firstly, the clinical heterogeneity of the patients resulted in variation within the dataset. The inclusion criteria was intentionally set less stringent in order to highlight the overall correlation of collagen and cell senescence across chronic wound patients. More wound tissues should be examined to support our findings. Secondly, patient’s matched unwounded arm skin or above knee tissues were used as reference sample. This is to provide a consistent background with similar biological makeups to base our assessments upon, substituting the risk of further biopsy injury to ulcerated regions of the limbs.

## Conclusion

Clinicians largely use physical assessments of the wound to determine which treatment methods to use. This often involves evaluating wounds without knowledge of the actual wound trajectory. The link established between presence of senescent cells and collagen area coupled with handheld two photon skin imaging could potentially serve as an indicator for the state of wound healing to denote if intervention is necessary.

## Supplementary Information

Supplementary Figure S1.
