# Supplementary material for: Extracellular matrix and cellular senescence in venous leg ulcers
Source: Sci Rep. 2021 Oct 11;11:20168. doi: 10.1038/s41598-021-99643-9 (PMC8505655; doi:10.1038/s41598-021-99643-9)
Supplement: Supplementary file 1 — Supplementary Figure S1. [file 41598_2021_99643_MOESM1_ESM.docx]

**Extracellular matrix and cellular senescence in venous leg ulcers**

Authors: Debbie X. E. Lim^1,2^, Toby Richards^3^, Muholan Kanapathy^4,5^, Thankiah Sudhaharan^6^, Graham D. Wright^6^, Anthony R. J. Phillips^7^ and David L. Becker*^1,2^

1. Lee Kong Chian School of Medicine, Nanyang Technological University Singapore, 11 Mandalay Road, Singapore
2. Skin Research Institute of Singapore, Agency for Science, Technology and Research, 11 Mandalay Road, Singapore
3. Faculty of Health and Medical Sciences, University of Western Australia, Perth, Australia
4. Division of Surgery & Interventional Science, University College London, United Kingdom
5. Department of Plastic and Reconstructive Surgery, Royal Free NHS Foundation Trust Hospital, London, United Kingdom
6. Microscopy Platform, Research Support Centre, Agency for Science, Technology and Research, Singapore
7. School of Biological Sciences, Auckland University, Auckland, New Zealand

**Correspondence author**

*Correspondence to David L. Becker

**Key words: Extracellular matrix, senescence, venous leg ulcer, diabetic foot ulcer, pressure ulcer, chronic wound.**

**Figure S1.**


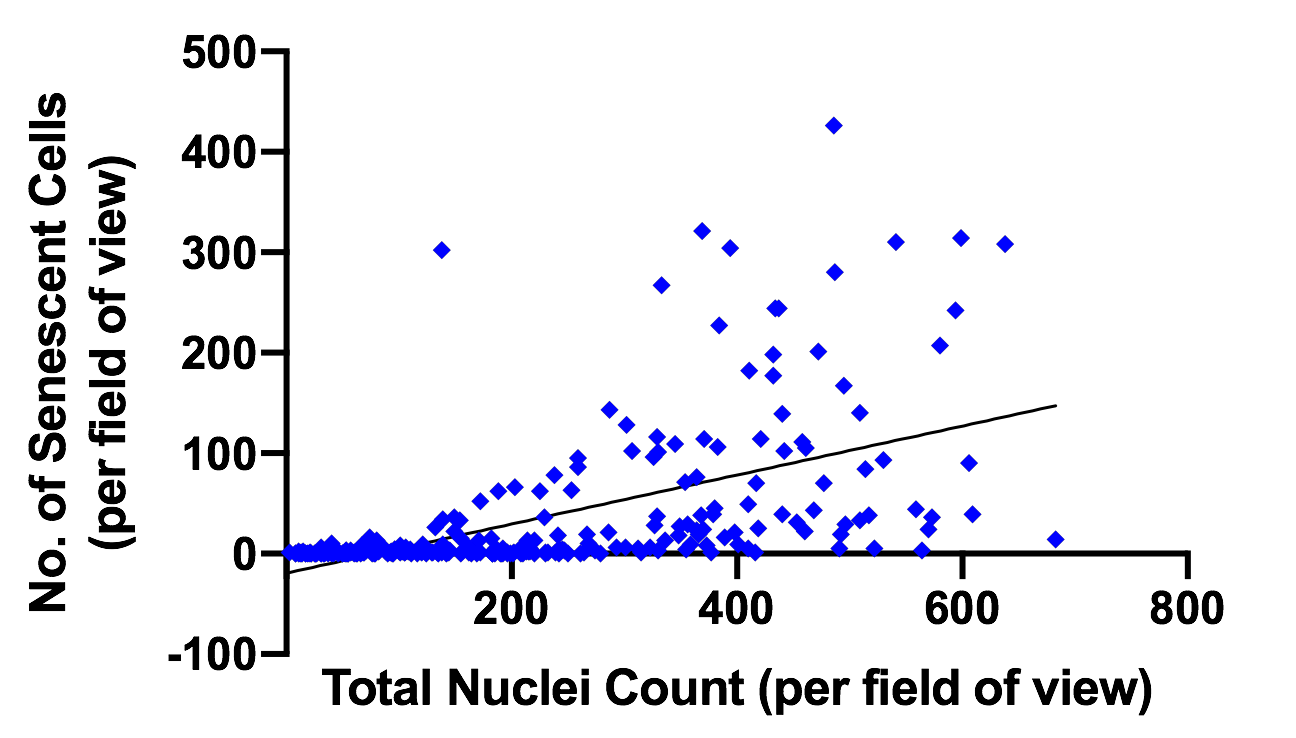


**Supplementary Information**

**Fig. S1** Plot of number of senescent cells against total nuclei count per field of view across all samples in our cohort.
